# Diversity in the Reproductive Modes of European *Daphnia pulicaria* Deviates from the Geographical Parthenogenesis

**DOI:** 10.1371/journal.pone.0020049

**Published:** 2011-05-31

**Authors:** France Dufresne, Silvia Marková, Roland Vergilino, Marc Ventura, Petr Kotlík

**Affiliations:** 1 Département de Biologie, Centre d'Études Nordiques, Université du Québec à Rimouski, Rimouski, Québec, Canada; 2 Laboratory of Fish Genetics, Department of Vertebrate Evolutionary Biology and Genetics, Institute of Animal Physiology and Genetics, Academy of Sciences of the Czech Republic, Liběchov, Czech Republic; 3 Biodiversity and Biogeodynamics Group, Center for Advanced Studies of Blanes, Spanish Research Council (CEAB-CSIC), Blanes, Girona, Catalonia, Spain; 4 Institut de Recerca de l'Aigua, Universitat de Barcelona, Barcelona, Catalonia, Spain; University of Cambridge, United Kingdom

## Abstract

**Background:**

Multiple transitions to obligate parthenogenesis have occurred in the *Daphnia pulex* complex in North America. These newly formed asexual lineages are differentially distributed being found predominantly at high latitudes. This conforms to the rule of geographical parthenogenesis postulating prevalence of asexuals at high latitudes and altitudes. While the reproductive mode of high-latitude populations is relatively well studied, little is known about the reproduction mode in high altitudes. This study aimed to assess the reproductive mode of *Daphnia pulicaria*, a species of the *D. pulex* complex, from high altitude lakes in Europe.

**Methodology/Principal Findings:**

Variation at eight microsatellite loci revealed that *D. pulicaria* from the High Tatra Mountains (HTM) had low genotype richness and showed excess of heterozygotes and significant deviations from Hardy-Weinberg expectations, and was thus congruent with reproduction by obligate parthenogenesis. By contrast, populations from the Pyrenees (Pyr) were generally in Hardy-Weinberg equilibrium and had higher genotypic richness, suggesting that they are cyclic parthenogens. Four lakes from lowland areas (LLaP) had populations with an uncertain or mixed breeding mode. All *D. pulicaria* had mtDNA ND5 haplotypes of the European *D. pulicaria* lineage. Pyr were distinct from LLaP and HTM at the ND5 gene. By contrast, HTM shared two haplotypes with LLaP and one with Pyr. Principal Coordinate Analysis of the microsatellite data revealed clear genetic differentiation into three groups. HTM isolates were intermediate to Pyr and LLaP, congruent with a hybrid origin.

**Conclusion/Significance:**

Inferred transitions to obligate parthenogenesis have occurred only in HTM, most likely as a result of hybridizations. In contrast to North American populations, these transitions do not appear to involve meiosis suppressor genes and have not been accompanied by polyploidy. The absence of obligate parthenogenesis in Pyr, an environment highly similar to the HTM, may be due to the lack of opportunities for hybridization.

## Introduction

Organisms that abandoned sex account for roughly 0.1% of all species [1], [2] but their very existence has long fascinated evolutionary biologists. It is intriguing that they do not outnumber sexual individuals despite a twofold transmission advantage. The lack of recombination is thought to render them vulnerable to extinction through the action of pathogens and environmental changes. Recent molecular studies have revealed that the majority of strictly asexual lineages are limited to a life span of 10,000 to 200,000 years [1]. Although the intrinsic constraints of asexuality may limit the evolutionary persistence time of individual asexual lineages, asexuality may persist in the long term if the rate of origin of asexuals is greater than the rate of extinction.

One pattern common to both asexual plants and animals is their more frequent distribution in extreme areas [3]. The prevalence of asexuals at high latitudes and altitudes and in extreme environments has long been recognised and called ever since geographical parthenogenesis [4]. Many hypotheses (not mutually exclusive) have been postulated to account for this pattern in nature. The relaxation of biotic pressures (fewer pathogens, competitors, predators) in extreme environments would allow asexuals to persist there [5]. Demographic hypotheses stipulate that asexuals are better colonizers than sexuals since a single individual can found a population [6]. Hence asexuals would preferentially colonize areas where sexuals are limited by their ability to find mates such as at the geographic edge of species ranges [7]. Asexuals would be better able to compete against sexuals in areas where the latter are in low density and inbred due to repeated bottlenecks [8]. The transmission advantages of asexuals relative to sexuals are thought to allow them to colonize new areas faster than sexuals [9]. Other hypotheses have singled out heterosis provided by the hybrid origins of many asexuals as the most important factor enabling them to invade extreme environments [10]. Understanding the reasons for the distinct distribution patterns of asexuals is a key step to understand their evolutionary fate.

Members of the *Daphnia pulex* complex comply with the geographical parthenogenesis pattern. The dominant and ancestral breeding mode in *Daphnia* is cyclical parthenogenesis that is an alternation between apomixis (eggs produced without fertilization) and sexual reproduction (through the production of resting eggs). Transitions to asexuality (obligate parthenogenesis) in *Daphnia* are only known in this species complex so that only four out of the 30 species in the *Daphnia* genus reproduce by obligate parthenogenesis [11]. Two of these species, *D. middendorffiana* and *D. tenebrosa*, are arctic endemics whereas the other two species, *D. pulex* and *D. pulicaria*, show variation in their breeding system. Arctic and subarctic populations (starting at 54°N) of *D. pulex* and *D. pulicaria* reproduce predominantly by obligate parthenogenesis whereas temperate populations reproduce either by obligate or cyclical parthenogenesis [12]. The switch to obligate parthenogenesis is thought to result from a dominant mutation, transmitted in a Mendelian fashion that suppress meiosis during resting egg formation in females but not during spermatogenesis in males such that males carrying the mutations can mate with females and the resulting progeny will be predominantly asexual [13]. The meiosis-suppressor gene is thought to have originated in eastern North America some 172,000 years ago and has been spreading westward [14]. As a result, northeastern populations of *D. pulex* are obligate asexuals, central populations (Ontario) are mixed, and northwestern and midwestern populations are sexuals [15]. By contrast, populations of *D. pulicaria* reproduce by obligate parthenogenesis in western North America and by cyclic parthenogenesis in eastern North America [16]. Obligately parthenogenetic and polyploid populations of *D. pulex* have recently been discovered in the Bolivian Andes [17]. A study of *D. pulex* in Europe has also revealed variation in breeding system and a pattern suggestive of geographical parthenogenesis with sexual populations in southern Sweden, mixed population occurring at intermediate latitudes in Scandinavia (60–61°N), and obligately parthenogenetic populations at higher latitudes [18]. However, we have little knowledge about the reproduction system of *D. pulicaria* in Europe, particularly those populations from the alpine lakes. Obligate parthenogenesis is thought to be advantageous in the arctic and alpine environments since the time allowed to reproduction is short and hence females that hatch from dormant eggs can readily invest their resources to produce dormant propagules rather than sparing a generation of parthenogenesis to produce males that would then mate with females to produce the resting eggs meiotically. It is noteworthy that four out of the six *Daphnia* species that inhabit arctic reproduce by obligate parthenogenenesis as opposed to 28 out of 30 temperate species that reproduce by cyclic parthenogenesis [11]. Interestingly, another cladoceran, *Holopedium*, reproduces by selfing or automixis in the arctic but by cyclic parthenogenesis in temperate zones, another beneficial way to escape cold and short growing seasons [19].

A recent study of *D. pulicaria* from alpine lakes in the High Tatra Mountains in Europe revealed high degree of heterozygosity at microsatellite loci, suggesting they may have been reproducing by obligate parthenogenesis [20], albeit a low number of individuals per populations had been analysed. Moreover, sequencing of the ND5 gene has shown that some individuals from the High Tatra Mountains and all sampled individuals from Pyrenees were closely allied to the *D. pulicaria* clade whereas most individuals from High Tatra Mountains and all from lowland areas of Europe belonged to the *D. tenebrosa* clade [20]. However, no microsatellite data have been recorded for *D. pulicaria* populations inhabiting Pyrenees and lowland Europe. Therefore the breeding system of these alpine populations is currently unknown.

This study aimed to: 1) assess the reproductive mode of *D. pulicaria* from 18 European high altitude and lowland populations to determine if they comply with the geographical parthenogenesis pattern, 2) get insights into the mode of origin of obligate parthenogenesis using information from mitochondrial sequences and clonal diversity patterns, and 3) determine if there have been multiple instances of transition to obligate parthenogenesis in these European lineages.

## Results

### Genetic diversity at microsatellites

**Figure 1:**
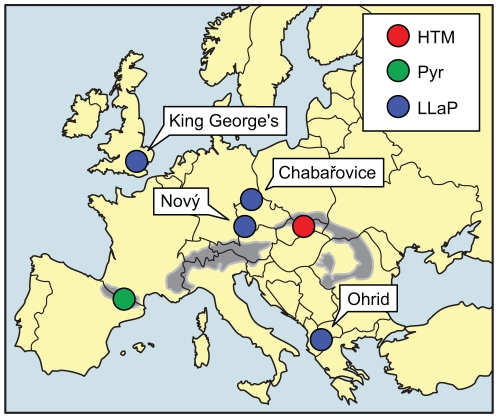
Geographic location of sampling sites. For the lowland populations (LLaP) each of the four sites is shown with a separate symbol while for the alpine populations single symbol is shown for each region (HTM and Pyr) where the sites were situated close to each other.

From 606 analyzed individuals collected in 18 water bodies representing the alpine and lowland populations of Europe (Figure 1) we obtained 561 isolates with complete genotype, which were used in subsequent statistical analyses (Table S1). A total of 64 alleles were identified at the eight microsatellite loci. Loci Dp519, Dp 514, and Dp502 had the lowest number of alleles (five) whereas locus Dp514alt was the most variable one, with 22 alleles. All analyzed individuals from two alpine lakes in the High Tatra Mountains (HTM; MHinc and ZelJ) were homozygous for the same allele at each locus, while isolates from all the other lakes and ponds were polymorphic.

The probability of identity statistics, P_(ID)sib_, calculated from the 561 reliable genotypes, predicted that the six most informative loci would be necessary, indicating that eight loci used in this study were sufficient to distinguish with 99% certainty between individuals that were not genetically identical (i.e. were not clones).

Combining the genotypes at the eight loci for each individual identified the total of 172 unique multilocus microsatellite genotypes (MLMGs). The lowest number of MLMGs was found in the HTM (one or two per lake) whereas the highest number (29) was found in lowland lakes and ponds (LLaP) in the pond Nový (Table S1). Sixty individuals shared the same MLMGs in HTM populations, 21 in Pyrenees (Pyr), and up to 15 in LLaP populations (Table 1). Generally highest genotypic richness (R) was observed in Pyr populations, whereas R values for HTM populations were very low, ranging from zero to 0.0017 (Table S1). Expected (He) as well as observed heterozygosity (Ho) assessed over all loci was generally highest in HTM populations and lowest in Pyr populations (Table S1), while the allelic richness was highest in LLaP populations.

**Table 1:** Probability of clonal identity in alpine (HTM and Pyr) and lowland (LLaP) populations where identical microsatellite genotypes (MLMGs) were encountered.

Region	Population	Repeated MLMGs	n	P_sex_	P_sex_ (F_is_)
HTM	ZelKriv	1	36	0.000	0.000
	ZelKriv	2	23	0.000	0.000
	CzarStaw	1	31	0.000	0.000
	CzarStaw	2	29	0.000	0.000
	VTSM	1	29	0.000	0.000
	VTSM	2	33	0.000	0.000
	MHinc	1	60	0.000	0.000
	ZelJ	1	30	0.000	0.000
Pyr	Estats	1	2	0.357	0.688
	Estats	2	3	0.154	0.623
	Sotllo	1	2	0.114	0.158
	Sotllo	2	2	0.477	0.707
	Redon	1	21	0.000	0.000
	Redon	2	2	0.002	0.037
	EG1	1	3	0.349	0.511
	EG1	2	2	0.549	0.638
	EG1	3	2	0.065	0.152
	EG3	1	2	0.276	0.261
	EG3	2	4	0.051	0.189
	EG3	3	1	0.321	0.305
	ENS	1	4	0.026	0.260
	ENS	2	3	0.072	0.422
	ENS	3	2	0.815	0.799
	ENS	4	2	0.283	0.469
	EPS	1	2	0.139	0.244
	ENG	1	5	0.928	0.836
	ENG	2	5	0.467	0.657
	ENG	3	2	0.697	0.776
	ENG	4	5	0.467	0.657
LLaP	KGeorge	1	11	0.000	0.000
	KGeorge	2	2	0.003	0.023
	KGeorge	3	3	0.000	0.000
	KGeorge	4	2	0.008	0.023
	KGeorge	5	5	0.000	0.000
	Chabarovice	1	2	0.053	0.219
	Chabarovice	2	3	0.000	0.012
	Chabarovice	3	15	0.000	0.000
	Ohrid	1	2	0.073	0.885
	Ohrid	2	9	2.153	0.002
	Ohrid	3	12	0.000	0.000

n, number of replicates; P_sex_, probability of clonal identity; P_sex_ (F_is_), corrected probability to consider possible departure from Hardy-Weinberg expectations.

### Genetic diversity at mitochondrial DNA

Restriction fragment length polymorphism (RFLP) analyses of the PCR product containing a part of the NADH dehydrogenase subunit 5 (ND5) gene indicated that all isolates could be assigned to European *D. pulicaria* in terms of mtDNA. The Eastern Nearctic *D. pulicaria* found in the HTM lakes in the year 2003 [20] thus was not recorded in the year 2005. A nucleotide sequence of 591bp of the ND5 gene was obtained for a representative isolate of each unique MLMG. There were 169 variable sites in the dataset, which revealed 23 distinct haplotypes. Levels of sequence polymorphism for each of the three geographic regions (HTM, Pyr and LLaP) are summarized in Table 2. The highest level of haplotype (h = 1.000) and nucleotide diversities (π = 0.031) were observed within HTM region, where each sequences were different from any other. The lowest values for the haplotype diversity (h = 0.569) was reported within LLaP and for nucleotide diversity (π = 0.005) within Pyr regions, respectively. Overall, nucleotide diversity among the 97 sequences was 0.020.

**Table 2:** Summary of sequence polymorphism for mtDNA ND5 gene.

Region	n	k	S	h±SD	π±SD
HTM	6	6	41	1.000±0.009	0.031±0.005
Pyr	27	9	17	0.778±0.004	0.005±0.002
LLaP	64	12	51	0.569±0.068	0.016±0.002
Total	97	23	52	0.787±0.038	0.020±0.001

n, number of sequences; k, number of haplotypes; S, number of variable sites; h, haplotype diversity; π, nucleotide diversity.

### Multivariate analyses of microsatellites

**Figure 2:**
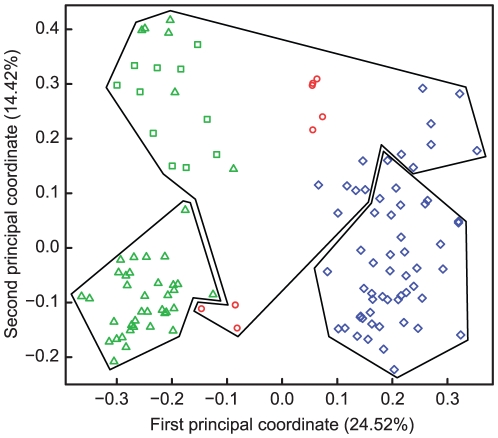
Principal Coordinate Analysis of microsatellite data. The first two principal coordinate axes are shown that represent 24% and 14% of the variation. K-means groups are represented with polygons surrounding isolates from Pyr lakes (green triangles) and ponds (green squares), from HTM (red circles), and from LLaP (blue diamonds).

Principal Coordinate Analysis (PCoA) revealed relatively good separation of populations from the geographic regions with the first two axes accounting for 24% and 14% of the variation (Figure 2). The calinski criterion obtained with CascadeKM indicated that three groups best represented our data. The first group included isolates from Pyr lakes, the second group included isolates from LLaP, and the third group included isolates from HTM, Pyr ponds and lakes, and LLaP. HTM isolates were intermediate those from Pyr lakes and LLaP. Furthermore, these results showed the presence of admixture between isolates from Pyr lakes and Pyr ponds (Figure 2).

### Mitochondrial DNA phylogeny

**Figure 3:**
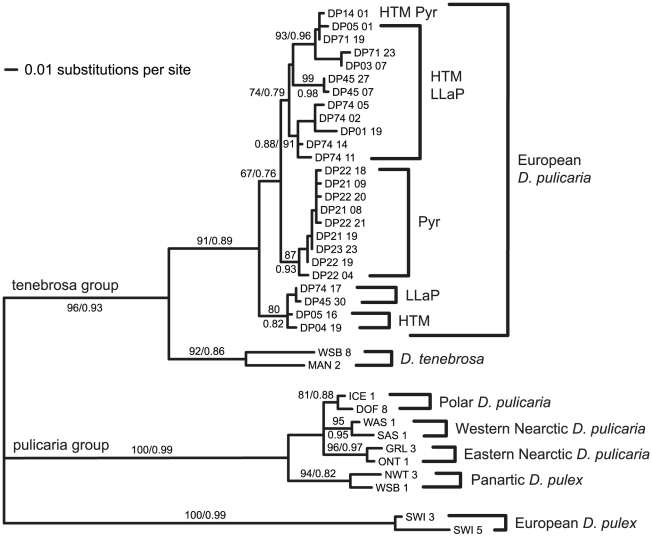
Maximum likelihood phylogeny of mtDNA haplotypes. Statistical support for the major clades is expressed as the percentage bootstrap proportions of 1000 replicates and as the SH-like approximate likelihood ratio probabilities.

**Figure 4:**
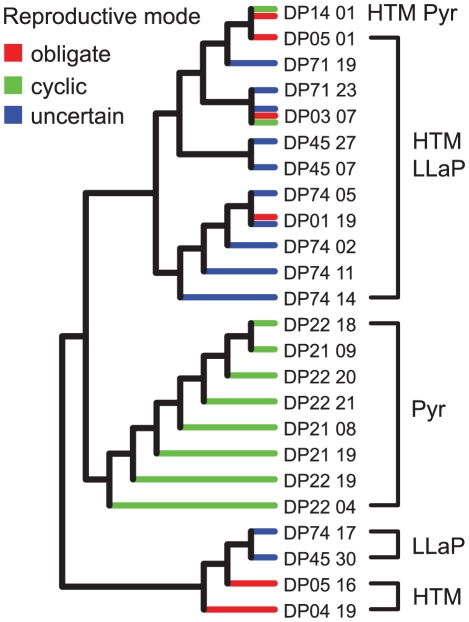
Phylogenetic relationships between obligate and cyclic parthenogens. The dendrogram of European mtDNA haplotypes is as in Figure 3 (branch lengths not to scale), with branches color-coded to indicate where the haplotype was found in an obligate parthenogenetic or cyclic parthenogenetic population or in a population with mixed or uncertain reproductive mode (see Discussion).

Combining new ND5 data with sequences from [11] resulted in a dataset containing 120 polymorphic sites, 100 of which were phylogenetically informative. Maximum-likelihood phylogenetic analysis of this data set resulted in a well-resolved tree (-Ln likelihood  =  2030.665; Figure 3), placing the all haplotypes from the three different regions in Europe (HTM, Pyr and LLaP) into the European *D. pulicaria* lineage (EuroPC), consistent with the RFLP analysis. Most of the haplotypes from Pyr cluster together and form a clade inside the EuroPC lineage. However, two Pyr sequences were identical with a HTM sequence (DP14_01 haplotype in Figure 3). HTM haplotypes were dispersed within LLaP haplotypes and, in addition, haplotypes DP01_19 and DP03_7 were shared with Lake Ohrid and with Lake Chabařovice and pond Nový, respectively, which suggested postglacial colonization of HTM and LLaP from the same source. Haplotypes of the obligate and cyclic parthenogens (see below) were interspersed in the phylogeny, and some haplotypes were found in obligate as well as cyclic populations (Figure 4).

### Genetic distances within and between regions

The highest sequence divergence at ND5 was found within HTM populations (3.2%) and the lowest among Pyr isolates (0.6%). HTM haplotypes were most divergent from Pyr haplotypes (3.0%) and more similar to LLaP haplotypes (2.7%; Table 3). At microsatellite loci, the highest genetic distance was found within Pyr region (51.3%), and Pyr populations were more similar to HTM (50.9%) than to the LLaP genotypes (45.2%; Table 3).

**Table 3:** Genetic distances between alpine (HTM and Pyr) and lowland (LLaP) populations based on mtDNA sequences (ND5 gene) and microsatellite data (MS).

Markers	Region	HTM	PyrM	LLaP
ND5	HTM	3.173	3.020	2.738
	Pyr	1.126	0.615	3.103
	LLaP	0.347	1.992	1.607
MS	HTM	50.387	50.897	47.123
	Pyr		51.317	45.184
	LLaP			45.266

Average ND5 pairwise model-corrected distance between groups is given above the diagonal and net distance below. Mean distance within each region is given along the diagonal. For MS, average Cavalli-Sforza's chord distance (D_SE_) between regions is given above the diagonal and within regions along the diagonal. All estimates are expressed as percentages.

### Evidence of clonal and sexual reproduction

#### High Tatra Mountains

Significant deviations from Hardy–Weinberg equilibrium (Hardy Weinberg equilibrium) and linkage disequilibrium (LD) were found in all HTM lakes when considering the entire dataset (F_is_^1^ from −0.486 to −1.000, P<0.001; r_d_^1^ from 0.003 to 0.007; Table S1) but not when including only a single individual for each genotype (Fis^2^ = −0.167; Table S1). r_d_^2^ values were not calculated in HTM, because of small number of isolates remained in the data set without multicopies (only one or two isolates). Genotype diversity ratio (genotypic diversity ratio) values varied from 0.667 to 0.738 and significant excess of heterozygotes was detected in all HTM lakes (P<0.001; Table S1). Significant P_sex_ values rejected the hypothesis that HTM individuals with identical MLMG have originated through distinct sexual reproductive events (P<0.001, Table 1).

#### Pyrenees

Pyr lakes did not show significant deviations from Hardy Weinberg equilibrium (after Bonferroni corrections) (F_is_^1^ from −0.293 to 0.106; Table S1). The score tests revealed heterozygote deficiency in a single Pyr population (Redon; P<0.001; Table S1). Four Pyr populations (Redon, EG1, ENS and ENG) showed significant LD values (P<0.01; Table S1) even after clone corrections (i.e. without multicopies). The ENG population had in addition a very low genotypic diversity ratio value (0.534) whereas the other Pyr populations had higher values (form 0.795 to 2.747; Table S1). Most of Pyr individuals had no significant P_sex_ values (most of populations exhibited P >0.05, except Redon and ENS populations; Table 1), hence suggesting that individuals with distinct MLMGs were likely to have originated through distinct sexual events.

#### Lowland ponds and lakes

Individuals from three LLaP lakes showed significant deviations from Hardy Weinberg equilibrium (F_is_^1^ from −0.435 to −0.794, P<0.001). Lake Ohrid had low genotypic diversity ratio value (0.665) but the other three LLaP populations had high genotypic diversity ratio values (from 0.891 to 1.035). Significant P_sex_ values were found in the majority of individuals with identical MLMG from King George's reservoir, lakes Chabařovice and Ohrid, suggesting that these individuals were not likely to have originated through distinct sexual events. All these three populations showed significant excess of heterozygotes. Pond Nový appeared to be inhabited by cyclic parthenogens as every sampled individual had a different MLMG and genotypic composition conformed to Hardy Weinberg equilibrium. The r_d_^1^ tests on LLaP populations (i.e. with multicopies) rejected the null hypothesis of recombination at the 0.001 level of significance for two populations; and at the 0.01 level for Lake Ohrid population, but the r_d_^2^ tests after clone correction did not do so for populations from King George's reservoir and Lake Ohrid. Taken as a whole, these analyses were consistent with a clonal reproduction regime, with occasional sexual reproduction in Lake Ohrid, King George's reservoir, and Lake Chabařovice but a sexual mode of reproduction in pond Nový.

## Discussion

### Genetic relationships of European *D. pulicaria* populations

The mtDNA analyses confirmed that all analysed *D. pulicaria* belonged to the European *D. pulicaria* clade. These results partly contrast with a previous study where some individuals from the High Tatra Mountains and Pyrenees were found to be closely related also to the Eastern lineage of North American *D. pulicaria* and were genetically distant from lowland populations [20]. The fact that the Eastern Nearctic *D. pulicaria* was not found in the present study despite a high sampling effort suggests that clones with this mtDNA lineage are rare (haplotypic diversity is high in HTM) or that their frequencies vary temporally. In addition, a new genotype was detected in Zelkriv lake in this study. These findings suggest that clonal composition and haplotypes change from year to year perhaps as a result of ecological changes. The North American lineages of the *D. pulex* complex have been quite successful in colonising new territories. An obligately parthenogenetic clone of Nearctic *D. pulex* has recently been found in Kenya where it has dispersed over distances of several hundreds of kilometers as a result of human-mediated transfer [21]. This lineage has also invaded naturally Northern Europe and is now well established in Scandinavia [22]. Natural dispersal of members of the *D. pulex* complex across the arctic is thought to have been facilitated by migratory birds with large arctic distribution [23]. European populations of *D. pulicaria* were structured in three distinct mtDNA clades, one consisting only of Pyr populations and two divergent ones including haplotypes from both HTM and LLaP. Populations from Pyr lakes were also distinct at the microsatellite level. By contrast, PCoA analyses revealed that HTM clustered with some LLaP and some Pyr isolates. A third cluster included solely LLaP.

### Reproductive mode in European *D. pulicaria*

Our study aimed to determine if *Daphnia* from alpine lakes in Europe comply with the geographical parthenogenesis pattern. The analyses of microsatellite markers revealed variation in reproductive mode in European populations of *D. pulicaria*. Several lines of evidence indicate that populations from the alpine lakes in HTM reproduce by obligate parthenogenesis. By contrast, although also inhabiting alpine environments, *D. pulicaria* from the Pyrenees appear to reproduce primarily by cyclic parthenogenesis. The mode of reproduction of LLaP populations was more difficult to decipher. Pond Nový population was in Hardy Weinberg equilibrium and linkage equilibrium and had high genotypic richness and genotypic diversity ratio values thus indicating that *Daphnia* from this pond reproduce by cyclic parthenogenesis. The three other LLaP populations were significantly in LD and were not in Hardy Weinberg equilibrium, and moreover had high genotypic diversity ratio thus their breeding system was perhaps mixed. LD after clone correction (r_d_^2^) was not significant in King George's and Ohrid populations, which suggested that recombination has not fully disrupted the associations between alleles caused by clonal reproduction, and the signal of sexual reproduction was apparent only when the data are clone-corrected. Mixed breeding system recorded in LLaP populations could be explained by occurrence of females recently hatched from resting eggs and females that were born to their asexual mother from eggs without fertilization. Previous work has revealed that lake *Daphnia* can forego sexual reproduction when there are little environmental cues to induce male production in the field [24] and hence lake *Daphnia* might not conform to Hardy Weinberg equilibrium despite reproducing by cyclic parthenogenesis. Mixed breeding system may also arise when apomictic clones invade sexual populations. Future studies should sample *Daphnia* shortly after hatching to help solve this question.

### Origin of obligate parthenogenesis in European *D. pulicaria*

Two different modes of origin of parthenogenesis are known in *Daphnia*: 1) mutation that disrupt meiosis in females as is found in North American *D. pulex* and *D. pulicaria* and 2) hybridization that disrupt meiosis due to chromosomal imbalances. Obligate parthenogenesis is unlikely to have arisen through the first mode as clonal diversity is very low in the HTM and high clonal diversity would be expected under a meiosis-suppressor gene model [16]. Several lines of evidence indicate that asexuality occurred through hybridization in HTM clones. First, the PCoA analyses on the microsatellite markers clearly showed that HTM clones occupy an intermediate position between Pyr and LLaP isolates. Second, HTM clones have very negative F_is_ values, and third, HTM clones have ND5 haplotypes that are found in two genetically divergent clusters as expected under reciprocal hybridization. Inferred transitions to obligate parthenogenesis have occurred on several occasions in European lineages of *D. pulicaria* (Figure 4). Although some LLaP populations appear mixed (composed of asexual and sexual individuals), HTM populations are the only ones where *Daphnia* were found to reproduce unambiguously by obligate parthenogenesis. Why have there been no transitions to obligate parthenogenesis in the Pyrenees populations? The genetic distinctiveness of Pyr populations suggests that they might have survived glaciation in the Pyrenees and hence quickly monopolised the habitat preventing other lineages from establishing there. Survival in a Pyrenean refugium has recently been suggested in the newt *Calotriton asper* [25] and in the bank vole *Myodes glareolus* [26], and is congruent with the high level of endemic taxa in the Northwestern Pyrenees. By contrast, ND5 analyses suggest that the HTM have recently been colonised by several distinct sources of *D. pulicaria* perhaps as a result of survival in different glacial refugia. Hybridization between refugial races is known to have generated parthenogenesis in a number of taxa [10]. Therefore, obligate parthenogens might occur solely in HTM because that area has been recolonised from multiple sources as opposed to Pyr populations.

### Geographical parthenogenesis

*Daphnia* from the High Tatra Mountains, in contrast to Pyr *Daphnia*, comply with geographical parthenogenesis, a differential distribution of asexuals at high altitude and latitudes [4]. *Daphnia* in the Bolivian Andes also reproduce by obligate parthenogenesis and are polyploids, a similar situation to what is found in the *D. pulicaria* clade from subarctic and arctic areas [27]. Polyploidy is often a confounding factor when considering explanations for the differential distributions of asexual lineages [28]. No evidence of polyploidy was found in HTM and Pyr *Daphnia*. Apparent transitions to obligate parthenogenesis have occurred a number of times in European *D. pulicaria* but it is restricted to HTM. One obvious advantage for asexual reproduction at high altitude lies in colonisation abilities, *i.e.* no need to find a partner, and in increased reproductive output. Furthermore, in areas where there is little time to complete a full reproductive life cycle, obligate parthenogenesis is advantageous since *Daphnia* can produce dormant eggs (the resistant egg stage) by mitosis without having to spare one generation in male production. Sexual and asexual lineages rarely occur in sympatry [29] and when they do they sometimes show microhabitat preferences [30]. It is not clear if obligate parthenogens are confined to HTM because they are better at colonizing remote areas or if they have higher fitness than cyclic parthenogens in HTM habitats.

### Conclusions

Transitions to obligate parthenogenesis have occurred multiple times in the *Daphnia pulex* complex. In North America, these transitions have arisen through a meiosis suppressor gene that has spread contagiously from a northeastern lineage. We suggest here that this mutation is not responsible for transitions to obligate parthenogenesis in European members of this complex. Rather, obligate parthenogenesis appears to have originated from hybridization between divergent mtDNA lineages of *Daphnia* from Pyr and LLaP and has led to the origin of a small number of clones confined to HTM.

## Materials and Methods

### Sample collection

A total of 606 individuals of *D. pulicaria* representing the alpine and lowland populations of Europe (Figure 1) were sampled (Table S1). Water fleas were caught in September and October of 2005 from five alpine lakes in the High Tatra Mountains (HTM) in the Western Carpathians of Slovakia and Poland. Three alpine lakes (Redon, Estats and Sotllo) and six alpine ponds (Table S1) in the Pyrenees in north-eastern Iberian Peninsula (Pyr) were sampled in September of 2005 and in July of 2007, respectively. Two lowland lakes and two ponds or reservoirs (LLaP) of the Czech Republic, Albania and United Kingdom (Table S1) were sampled in May of 2003 and 2004 and in July of 2004 and 2005. *Daphnia* were collected by plankton net tows from an inflatable boat in the lake center or by horizontal sweeps on the lakeshore, and were stored in 95% ethanol at 4°C until analysis.

### Microsatellite genotyping

DNA was extracted using the IsoQuick Nucleic Acid Extraction Kit (ORCA research, USA) following the manufacturer's instruction. Genetic variation was assessed using eight microsatellite loci Dp183, Dp502, Dp512, Dp514, Dp514alt, Dp519, Dp522 and Dp523 described by [31]. Loci Dp183, Dp512, Dp519, Dp522 and Dp523 consisted of dinucleotide motifs and loci Dp502, Dp514 and Dp514alt contained trinucleotide repeats. Forward primers were end-labeled with fluorescent dyes (Life Technologies, Univ. of Oklahoma, USA). Polymerase chain reaction (PCR) amplifications were carried out as described by [31] and at the *Daphnia* Genomics Consortium website (http://daphnia.cgb.indiana.edu). Sizes of amplified microsatellite alleles were scored using a denaturing polyacrylamide gel and visualized using a FM-BIO III scanner (Hitachi).

### Mitochondrial DNA sequencing and RFLP analysis

A 711-bp fragment including part of the gene coding for the NADH dehydrogenase subunit 5 (ND5) was amplified by PCR from genomic DNA by using a newly designed forward (5′-AAA CCT CTA AAB TTC YKA RCT- 3′) and reverse primer (5′-CAT RTT YAT RTC RGG GGT TGT- 3′). Each PCR (total volume of 25 µl) was composed of 1x PPP Master Mix (Top Bio, Prague, Czech Republic) and 0.5 mM or 1 mM primers. The PCR amplification consisted of an initial denaturing at 94°C for 2 min followed by 38 cycles of denaturing at 94°C for 40 s, annealing at 51°C for 60 s, and extension at 72°C for 90 s with a final extension period of 15 min at 72°C.

The ND5 PCR product from a representative isolate of each multilocus microsatellite genotype (MLMG; see below) was then sequenced. For this, PCR products were purified using the QIAquick PCR Purification Kit (Qiagen, Valencia, CA, USA). Sequence analysis was carried out on a 3730xl DNA analyzer (Applied Biosystems, Forster City, CA, USA). Sequences were aligned manually and all polymorphisms were additionally checked by visual inspection of automated sequencer chromatograms. Nucleotide sequences of each unique haplotype will have been deposited in the GenBank database (JF815581-JF815604).

**Figure 5:**
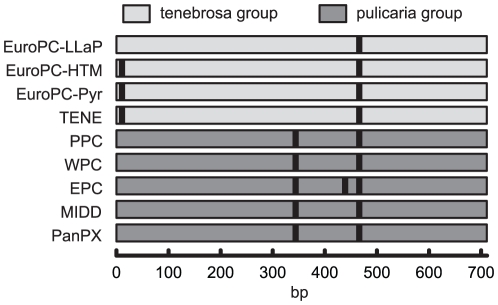
Restriction map of the mtDNA ND5 amplicon. The *Apo*I site at position 344 distinguishes species [11] of the *tenebrosa* group (European *Daphnia pulicaria*, EuroPC; and *D. tenebrosa*, TENE) from those of the *pulicaria* group (polar, PPC; western, WPC; and eastern *D. pulicaria*, EPC; *D. middendorffiana*, MIDD; and panarctic *D. pulex*, PanPX). Some lineages yield short fragments under 100 bp in size difficult to visualize using agarose gels but this does not affect scoring the individuals as either tenebrosa or pulicaria group.

PCR products of all the remaining isolates were subjected to RFLP analysis in order to verify that they belonged to European *D. pulicaria* and not to other species that exist within the *D. pulex* complex [11]. Species delimitation and taxonomy is problematic in the *D. pulex* complex. North American and European populations of *D. pulicaria* have the same species name despite the fact that these two groups should not belong to the same species since they are genetically distinct [11]. This study focuses on European *D. pulicaria*. ND5 haplotypes from other lineages are included for genetic comparisons. A restriction map was generated using the CLC Free Workbench 4.6.2 (CLC Bio A/S) based on available ND5 sequences of all mtDNA clades of the *D. pulex* complex [11], [20]. The six-base cutting endonuclease *Apo*I was predicted to yield group-specific RFLP profiles following digestion of the ND5 amplicon due to a diagnostic cleavage site present only in the *pulicaria* group of species of the complex that is absent in the *tenebrosa* group (Figure 5), which unambiguously identified each isolate to either the *tenebrosa* or the *pulicaria* group [11]. The PCR products were digested with ApoI following the manufacturer's (New England Biolabs, Ipswich, MA) specification. *Daphnia tenebrosa* (clade TENE) does not occur in Europe except in the Arctic [23], and the European *D. pulicaria* (EuroPC) is therefore the only representative of the *tenebrosa* group in the area covered by this study. It was therefore safe to assign all isolates showing the RFLP profile specific for the *tenebrosa* group (Figure 5) as belonging to EuroPC.

### Power of multilocus genotyping

In order to determine if a sufficient number of microsatellite loci have been scored to distinguish between individuals with different genotypes, we estimated the probability of identity, P_(ID)_, which is the probability that two individuals in a population will have the same genotype at a defined number of multiple loci [32]. Because strong population substructure in the data can cause substantial bias in P_(ID)_ due to individuals within each subpopulation being more closely related [32], we used an estimate of P_(ID)_ among siblings, P_(ID)sib_ [32], to provide the upper limit of the possible ranges of P_(ID)_ and thus the most conservative number of loci required to resolve all individuals with confidence [32]. P_(ID)sib_ values were estimated using the program GIMLET 1.3.3 [33].

### Genetic diversity

#### Microsatellites

In order to summarize the variation at the microsatellite loci, we calculated the number of distinct MLMGs detected per population (G), as well as measures of genotypic (R) and allelic richness (AR), expected (He) and observed heterozygosity (Ho), and the inbreeding coefficient (F_is_). The parameters were estimated by the computer programs GENCLONE [34], MSA [35] and GENETIX 4.04 [36]. Significance of F_is_ values was determined with 10 000 randomizations. F_is_ were calculated from the entire dataset and also when considering only the first individual of each genotype of the dataset. Two variants of exact test of Hardy Weinberg equilibrium were conducted for each population by using Genepop 4.0 [37], which both assume the same null hypothesis (random union of gametes) but differ in the construction of the rejection zone. The score tests (U tests) were run, to assume heterozygote excess or heterozygote deficiency as the alternative hypothesis to panmixia [38]. The Markov chain algorithm to estimate without bias the exact P-value of this test [39] was conducted with 1,000 batches of 20,000 iterations following 20,000 dememorization steps. We also assessed whether random sexual reproduction occurred by estimating the index of multilocus linkage disequilibrium (r_d_) using the MULTILOCUS software, version 1.3 [40]. This index is based on the index of association (IA), allowing one to test for random recombination between pairs of loci by comparing the observed and expected variance of genetic distance between all pairs of individuals [40]. Departure from the null hypothesis (no linkage disequilibrium, i.e. r_d_ = 0) was assessed by permuting alleles between individuals independently for each locus (1000 permutations).

#### Mitochondrial ND5 gene

Several measures of polymorphism were calculated to quantify the level of genetic variation at the ND5 gene within geographic regions, using DnaSP, version 4.90.1 [41]. For each region (HTM, Pyr and LLaP), the number of haplotypes (k), number of polymorphic sites (S), haplotype diversity (h), mean number of nucleotide differences (nucleotide diversity, π) were estimated.

### Genetic relationships

#### Multivariate analysis of microsatellites

To assess genetic relatedness among isolates from different populations and regions, alleles were transformed to produce a binary matrix (0 for absence and 1 for presence). Nei's index [42] was calculated using Phyltools version 1.32 [43] to construct a distance matrix between all the genotypes. This distance was chosen in preference to other distance measures, as it does not use shared absence of an allele as a shared characteristic [44]. A Principal Coordinate Analysis (PCoA) [45] was conducted on the distance matrix to represent affinities between genotypes using the module pco of the R software Version 4.0 [46], [47]. To choose the most probable number of clusters represented by our distance matrix, we used an iterative method, CascadeKM, implemented in the vegan package available with the R software. We tested how many clusters best represented our distance matrix. The “calinski” criterion [48] available in the module CascadeKM was used to search the best partitions that represent our data. After selecting the best number of clusters, K-means algorithm [49] was applied to the different distance matrices using kmeans module implemented in the stats package available with the R software.

#### MtDNA haplotype phylogeny

Phylogenetic relationships among the ND5 sequences were reconstructed using the maximum likelihood optimality criterion. We also included 12 homologous sequences of members of the *D. pulex* complex including four constituent clades of the *pulicaria* group (termed polar, western and eastern lineage of Neartic *D. pulicaria*, and panartic *D. pulex*) and *D. tenebrosa* of the *tenebrosa* group described by [11]. Sequences of European *D. pulex* were used to root the trees. The TPM3uf+I model of sequence evolution (three-parameter model with unequal base frequencies and a proportion of invariable sites) [50] was determined to be the appropriate model according to the Akaike information criterion implemented in the ModelTest program, version 0.1.1 [51]. Maximum-likelihood phylogenetic analyses were performed using the BEST approach implemented in PhyML 3.0.1, which combines the nearest-neighbour interchanges with the subtree pruning and regrafting algorithms to maximize tree likelihood, and using the TPM3uf+I model, with the base frequencies A, 0.18; C, 0.21; G, 0.20; T, 0.41; and the proportion of invariable sites equal to 0.549. To quantify the confidence in the partitioning within the ML tree we used the approximate likelihood ratio test (aLRT) implemented in PhyML and we also performed the nonparametric bootstrap test [52] with 1000 replications.

#### Genetic distances between regions

Pairwise TPM3uf+I model-corrected distances between ND5 sequences were estimated with the PAUP* software package, version 4.0b10 D [53]. Pairwise Cavalli-Sforza's chord distances, D_SE_ [54], between populations were calculated from microsatellite data with GENDIST module of PHYLIP, version 3.69 [55]. The resulting distance matrices were imported into MEGA, version 4.0.2 [56], and the average distances between and also within regions (HTM, Pyr and LLaP) calculated.

### Detection of clonal reproduction

The genotyping with eight independent microsatellite markers allows the assignment of isolates into several groups of multilocus microsatellite genotypes, MLMGs. In order to determine if these MLMGs resulted from clonal or sexual events, we calculated P_sex_ that is the probability that individuals with identical MLMGs originate from distinct sexual reproductive events [57]. Below a threshold value fixed at 0.01, identical MLMGs may be considered as belonging to the same clone (or to reproduce clonally). Estimates of P_sex_ are derived on the basis of allelic frequencies according to the round robin method with a sub-sampling approach to limit the overestimation of the rare alleles [57]. Allelic frequencies for each locus are estimated on the basis of a sample pool composed of all the MLMGs discriminated, while excluding the loci for which allelic frequency is estimated [57]. In addition to P_sex_ we also estimated the probability P_sex_ (F_is_) to consider possible departures from Hardy Weinberg equilibrium in order to obtain a more conservative estimate of P_sex,_ [57]. All calculations were performed using the software GENCLONE [34]. In addition, we also used the classical procedure of [16] to determine if *Daphnia* populations reproduce by obligate or by cyclic parthenogenesis: we calculated the genotype diversity ratio (genotypic diversity ratio), which is the ratio of the number of observed genotypes to the number of genotypes expected under independent segregation of loci. Populations with genotypic diversity ratio smaller than 0.75 and out of Hardy Weinberg equilibrium are regarded as obligatory asexual whereas those with genotypic diversity ratio greater than 0.75 and in Hardy Weinberg equilibrium are considered cyclic parthenogens. Populations with genotypic diversity ratio of 0.75 and significantly out of Hardy Weinberg equilibrium have uncertain or mixed breeding systems.

## Supporting Information

Table S1 Population information and statistics based on microsatellite data.
(PDF)

## References

[1] Bell G (1982). The masterpiece of nature: The Evolution and Genetics of Sexuality..

[2] Schurko A, Neiman M, Logsdon JM (2009). Signs of sex: what we know and how we know it.. TREE.

[3] Bierzychudek P (1985). Patterns in plant parthenogenesis.. Experientia.

[4] Vandel A (1928). La parthénogénèse géographique. Contribution à l'étude biologique et cytologique de la parthénogénèse naturelle. Bull. Biol.. Français Belgique.

[5] Glesener RR, Tilman D (1978). Sexuality and the components of environmental uncertainty: Clues from geographic parthenogenesis in terrestrial animals.. Am. Nat.

[6] Cuellar O (1977). Intraclonal histocompatibility in a parthenogenetic lizard: evidence of genetic homogeneity.. Science.

[7] Peck JR, Yearsley JM, Waxman D (1998). Explaining the geographic distributions of sexual and asexual populations.. Nature.

[8] Haag CR, Ebert D (2004). A new hypothesis to explain geographic parthenogenesis.. Ann Zool Fenn.

[9] Law JH, Crespi BJ (2002). The evolution of geographic parthenogenesis in *Timema* walking-sticks.. Mol Ecol.

[10] Kearney M (2005). Hybridization, glaciation and geographical parthenogenesis.. TREE.

[11] Colbourne JK, Crease TJ, Weider LJ, Hebert PDN, Dufresne F (1998). Phylogenetics and evolution of a circumarctic species complex (Cladocera: *Daphnia pulex*).. Biol J Linn Soc B.

[12] Beaton MJ, Hebert PDN (1988). Geographical parthenogenesis and polyploidy in *Daphnia pulex*.. Am Nat.

[13] Innes DJ, Hebert PDN (1988). The origin and genetic basis of obligate parthenogenesis in *Daphnia pulex*.. Evolution.

[14] Paland S, Colbourne JK, Lynch M (2005). Evolutionary history of contagious asexuality in *Daphnia pulex*.. Evolution.

[15] Hebert PDN, Finston TL (2001). Macrogeographic patterns of breeding system diversity in the *Daphnia pulex* group from the United States and Mexico.. Heredity.

[16] Hebert PDN, Swartz SS, Ward RD, Finston TL (1993). Macrogeographic patterns of breeding system diversity in the *Daphnia pulex* group. I. Breeding systems of Canadian populations.. Heredity.

[17] Aguilera X, Mergeay J, Wollebrants A, Declerck S, De Meester L (2007). Asexuality and polyploidy in *Daphnia* from the tropical Andes.. Limnol Oceanogr.

[18] Ward RD, Bickerton MA, Finston F, Hebert PDN (1994). Cline in breeding systems and ploidy levels in European populations of *Daphnia pulex*.. Heredity.

[19] Hebert PDN, Rowe CL, Adamowicz SJ (2007). Life at low temperatures: A novel breeding-system adjustment in a polar cladoceran.. Limnol Oceanogr.

[20] Marková S, Dufresne F, Rees DJ, Černý M, Kotlík P (2007). Cryptic intercontinental colonization in water fleas *Daphnia pulicaria* inferred from phylogenetic analysis of mitochondrial DNA variation.. Mol Phylogenet Evol.

[21] Mergeay J, Verschuren D, De Meester L (2006). Invasion of an asexual American water flea clone throughout Africa and rapid displacement of a native sibling species.. Proc Roy Soc Lond B.

[22] Pálsson S (2000). Microsatellite variation in *Daphnia pulex* from both sides of the Baltic Sea.. Mol Ecol.

[23] Weider LJ, Hobæk A, Colbourne JK, Crease TJ, Dufresne F (1999). Holarctic phylogeography of an asexual species complex I. Mitochondrial DNA variation in arctic *Daphnia*.. Evolution.

[24] Černý M, Hebert PDN (1993). Genetic diversity and breeding system variation in *Daphnia pulicaria* from North American lakes.. Heredity.

[25] Mila B, Carranza S, Olivier G, Clobert J (2010). Marked genetic structuring and extreme dispersal limitation in the Pyrenean brook newt *Calotriton asper* (Amphibia: Salamandridae) revealed by genome-wide AFLP but not mtDNA.. Mol Ecol.

[26] Deffontaine V, Ledevin R, Fontaine MC, Quéré J-P, Renaud S (2009). A relict bank vole lineage highlights the biogeographic history of the Pyrenean region in Europe.. Mol Ecol.

[27] Dufresne F, Hebert PDN (1997). Pleistocene glaciations and polyphyletic origins of polyploidy in an arctic cladoceran.. Proc Roy Soc Lond B.

[28] Dufresne F, Hebert PDN (1998). Temperature-related differences in life-history characteristics between diploid and polyploid clones of the *Daphnia pulex* complex.. Écoscience.

[29] Suomalainen E, Saura A, Lokki J (1987). Cytology and Evolution in Parthenogenesis..

[30] Lehto MP, Haag CR (2010). Ecological differentiation between coexisting sexual and asexual strains of *Daphnia pulex*.. J Anim Ecol.

[31] Colbourne JK, Robison B, Bogart K, Lynch M (2004). Five hundred and twenty–eight microsatellite markers for ecological genomic investigations using *Daphnia*.. Mol Ecol Notes.

[32] Waits LP, Luikart G, Taberlet P (2001). Estimating the probability of identity among genotypes in natural populations: cautions and guidelines.. Mol Ecol.

[33] Valière N (2002). GIMLET a computer program for analysing genetic individual identification data.. Mol Ecol Notes.

[34] Arnaud-Haond S, Belkhir K (2007). GENCLONE 1.0: a new program to analyse genetics data on clonal organisms.. Mol Ecol Notes.

[35] Dieringer D, Schlötterer C (2003). Microsatellite Analyzer (MSA): a platform independant analysis tool for large microsatellite data sets.. Mol Ecol Notes.

[36] Belkhir K, Borsa P, Goudet J, Chikhi L, Bonhomme F (2003). http://www.genetix.univ-montp2.fr/genetix/genetix.htm.

[37] Rousset F (2008). Genepop: a complete re-implementation of the genepop software for Windows and Linux.. Mol Ecol Res.

[38] Raymond M, Rousset F (1995). GENEPOP (version 1.2): population genetics software for exact tests and ecumenicism.. J Hered.

[39] Guo SW, Thompson EA (1992). Performing the exact test of Hardy–Weinberg proportion for multiple alleles.. Biometria.

[40] Agapow PM, Burt A (2001). Indices of multilocus linkage disequilibrium.. Mol Ecol Notes.

[41] Rozas J, Sanchez-Delbarrio JC, Messeguer X, Rozas R (2003). DnaSP, DNA polymorphism analyses by the coalescent and other methods.. Bioinformatics.

[42] Nei M, Li WH (1979). Mathematical model for studying genetic variation in terms of restriction endonucleases.. Proc Natl Acad Sci.

[43] Buntjer JB (2001). Phylogenetic Computer Tools (PhylTools). Version 1.32 for Windows..

[44] Legendre P, Legendre L (1998). Numerical Ecology, 2nd edn..

[45] Gower JC (1966). Some distance properties of latent root and vector methods used in multivariate analysis.. Biometrika.

[46] Ihaka R, Gentleman R (1996). R: a language for data analysis and graphics.. Journal of Computational and Graphical Statistics.

[47] R Development Core Team (2004). http://www.R-project.org.

[48] Calinski T, Harabasz J (1974). A dendrite method for cluster analysis.. Communications in Statistics.

[49] Hartigan JA, Wong MA (1979). Algorithm AS 136: A K-means clustering algorithm.. Journal of the Royal Statistical Society Series C (Applied Statistics).

[50] Posada D, Baxevanis A, Davison DB, Page RDM, Petsko GA, Stein LD, Stormo GD (2003). Using Modeltest and PAUP* to select a model of nucleotide substitution.. Current Protocols in Bioinformatics.

[51] Posada D (2008). JModelTest: Phylogenetic Model Averaging.. Mol Biol Evol.

[52] Felsenstein J (1985). Confidence-limits on phylogenies - an approach using the bootstrap.. Evolution.

[53] Swofford DL (2003). PAUP*.Phylogenetic Analysis Using Parsimony and Other Methods), Version 4.0..

[54] Cavalli–Sforza LL, Edwards AWF (1967). Phylogenetic analysis: models and estimation procedures.. Evolution.

[55] Felsenstein J (2005). PHYLIP (Phylogeny Inference Package) version 3.6..

[56] Tamura K, Dudley J, Nei M, Kumar (2007). MEGA 4: molecular evolutionary genetics analysis (MEGA) software version 4.0.. Mol Biol Evol.

[57] Arnaud-Haond S, Duarte CM, Alberto F, Serrao EA (2007). Standardizing methods to address clonality in population studies.. Mol Ecol.

