# Supplementary material for: Diversity in the Reproductive Modes of European Daphnia pulicaria Deviates from the Geographical Parthenogenesis
Source: PLoS One. 2011 May 31;6(5):e20049. doi: 10.1371/journal.pone.0020049 (PMC3104988; doi:10.1371/journal.pone.0020049)
Supplement: Table S1 — Population information and statistics based on microsatellite data. (PDF) [file pone.0020049.s001.pdf]

Table S1. Population information and statistics based on microsatellite data.

Population (code)	*Lat*	*Long*	*Alt (m)*	*n*	*Ngen*	*G*	*R*	*Var*	*AR*	H_e	H_o	F_{is}^1	F_{is}^2	*HE (P)*	*HD (P)*	r_d^1	r_d^2	*GDR*
**High Tatra Mts. (HTM)**																		
Zelené Krivánske (ZelKriv)	49.159	20.008	2017	60	59	2	0.017	35.753	2.875	0.586	0.875	-0.486***	-0.167	0.000	1.000	0.003***	N/A	0.734
Czarny Staw pod Rysmi (CzarStaw)	49.189	20.078	1580	60	60	2	0.017	32.677	2.875	0.594	0.875	-0.468***	-0.167	0.000	1.000	0.007***	N/A	0.736
Vyšné Temnosmrečianske (VTSM)	49.189	20.038	1716	62	62	2	0.016	15.776	2.875	0.593	0.875	-0.469***	-0.167	0.000	1.000	0.005***	N/A	0.738
Malé Hincovo (MHinc)	49.174	20.057	1923	60	60	1	0.000	42.267	1.875	0.438	0.875	-1.000***	N/A	0.000	1.000	N/A	N/A	0.667
Zelené Javorove (ZelJ)	49.206	20.142	1811	30	30	1	0.000	90.808	1.875	0.438	0.875	-1.000***	N/A	0.000	1.000	N/A	N/A	0.667
**Pyrenees (Pyr)**																		
Estats (Estats)	42.659	1.391	2470	30	27	24	0.885	8.027	2.250	0.271	0.329	-0.195*	-0.181*	0.028	0.970	0.024	0.005	1.064
Sotllo (Sotllo)	42.652	1.385	2346	22	16	14	0.867	7.174	1.750	0.242	0.305	-0.226*	-0.248*	0.018	0.983	0.043	0.022	0.795
Redon (Redon)	42.642	0.780	2240	32	29	8	0.250	34.234	2.500	0.284	0.259	0.106	0.318	1.000	0.000	0.069***	0.007***	2.202
Estanyet de Gerber 1 (EG1)	42.621	0.992	2341	22	15	11	0.714	1.674	2.250	0.201	0.233	-0.126	-0.042	0.217	0.782	0.051***	0.014***	0.937
Estanyet de Gerber 2 (EG2)	42.621	0.991	2341	22	14	14	1.000	3.269	2.375	0.283	0.295	-0.004	-0.004	0.380	0.625	0.011	0.122	0.862
Estanyet de Gerber 3 (EG3)	42.622	0.991	2341	22	18	13	0.706	3.852	2.000	0.195	0.229	-0.145	-0.103	0.491	0.503	0.033	0.012	2.164
Estany Negre de Salenques (ENS)	42.604	0.693	2590	22	18	8	0.412	1.000	1.750	0.196	0.208	-0.034	0.133	0.998	0.002	0.084**	0.099**	2.747
Estany del Port de Salenques (EPS)	42.607	0.683	2660	20	17	16	0.938	11.495	2.000	0.275	0.324	-0.146	-0.147	0.085	0.916	-0.020	0.036	0.875
Estang Negre de Gerber (ENG)	42.616	0.990	2440	20	17	4	0.188	0.927	1.375	0.067	0.088	-0.293*	-0.200	0.046	1.000	0.084**	N/A	0.534
**European lowland lakes and ponds (LLaP)**																		
King George’s reservoir (KGeorge)	51.452	-0.502	17	30	29	11	0.357	24.691	2.750	0.386	0.582	-0.493***	-0.415***	0.000	1.000	0.069***	-0.046	0.936
pond Nový (Novy)	49.437	13.785	429	30	29	29	1.000	46.415	4.500	0.497	0.509	-0.007	-0.007	0.456	0.505	0.031	0.015	0.891
Lake Chabařovice (Chabarovice)	50.654	13.944	137	32	32	15	0.452	46.096	3.250	0.381	0.551	-0.435***	-0.257***	0.000	1.000	0.037***	0.013***	1.035
Lake Ohrid (Ohrid)	41.020	20.700	695	30	29	9	0.286	4.273	2.250	0.419	0.754	-0.794***	-0.705***	0.000	1.000	0.027**	0.011	0.665

Lat, latitude; Long, longitude; Alt, altitude; n, number of individuals; Ngen, number of individuals with complete genotype; G, number of distinct multilocus genotypes; R, genotypic richness; Var, variance in allele size; AR, allelic richness; H_e, expected heterozygosity; H_o, observed heterozygosity; Fis, inbreeding coefficient calculated using dataset with multicopies (F_{is}^1) and without multicopies (F_{is}^2); P, probabilities for the exact test of Hardy-Weinberg equilibrium assuming, respectively, heterozygote excess (HE) and deficiency (HD) as the alternative hypothesis to panmixia. Multilocus linkage disequilibrium (rd) tests were performed on dataset with multicopies (rd^1) and without multicopies (rd^2). GDR, genotypic diversity ratio. Statistical significance, *P < 0.05; **P < 0.01; ***P < 0.00.
